# SATB2-Nanog axis links age-related intrinsic changes of mesenchymal stem cells from craniofacial bone

**DOI:** 10.18632/aging.101041

**Published:** 2016-09-14

**Authors:** Peipei Zhou, Geng Wu, Ping Zhang, Rongyao Xu, Jie Ge, Yu Fu, Yuchao Zhang, Yifei Du, Jinhai Ye, Jie Cheng, Hongbing Jiang

**Affiliations:** ^1^ Jiangsu Key Laboratory of Oral Diseases, Nanjing Medical University, 210029 Nanjing, China; ^2^ Department of Oral and Maxillofacial Surgery, Affiliated Hospital of Stomatology, Nanjing Medical University, 210029 Nanjing, China

**Keywords:** aging, bone mesenchymal stem cells, pluripotency, bone loss, cytotherapy

## Abstract

Bone mesenchymal stem cells (BMSCs) senescence contributes to age-related bone loss. The alveolar bone in jaws originates from neural crest cells and possesses significant site- and age-related properties. However, such intrinsic characteristics of BMSCs from alveolar bone (AB-BMSCs) and the underlying regulatory mechanisms still remain unknown. Here, we found that the expression of special AT-rich binding protein 2 (SATB2) in human AB-BMSCs significantly decreased with aging. SATB2 knockdown on AB-BMSCs from young donors displayed these aging-related phenotypes in vitro. Meanwhile, enforced SATB2 overexpression could rejuvenate AB-BMSCs from older donors. Importantly, satb2 gene- modified BMSCs therapy could prevent the alveolar bone loss during the aging of rats. Mechanistically, the stemness regulator Nanog was identified as the direct transcriptional target of SATB2 in BMSCs and functioned as a downstream mediator of SATB2. Collectively, our data reveal that SATB2 in AB-BMSCs associates with their age-related properties, and prevents AB-BMSCs senescence via maintaining Nanog expression. These findings highlight the translational potential of transcriptional factor-based cellular reprogramming for anti-aging therapy.

## INTRODUCTION

The adult skeleton is a high-renewal organ undergoing continuous bone remodeling via bone formation by osteoblasts and bone resorption by osteoclasts. In aging population, osteoporosis and associated bone fractures often occur when bone resorption excessively exceeds formation, thus leading to morbidity and heavy socioeconomic burden [1]. Accumulating evidence suggests that age-dependent decline in the quantity and functions of bone mesenchymal stem cells (BMSCs) are largely attributed to bone loss in the aged or postmenopausal skeleton [2]. Age-related decrease of BMSCs quantity is the result of their lifespan decline and responsible for impaired bone regeneration [3]. On the other side, the intrinsic properties of BMSCs such as senescence, osteogenic/adipogenic differentiation potential, and osteoclastogenesis activity were markedly changed during aging process and also associated with skeletal aging and bone loss [4, 5]. These findings provide strong evidence that age-related changes of BMSCs compromise bone remodeling especially the bone regenerative potential, and contribute to bone loss.

As a biological aging “window” of craniofacial bone, the alveolar bone displays key associations between age-related osteoporosis and tooth loss or alveolar atrophy [6, 7]. Mounting evidence has established that craniofacial bone arises from neural crest cells of neuroectoderm germ layer while axial and appendicular bone is from mesoderm [8]. Our previous findings and others have revealed that BMSCs from craniofacial bone exhibit some unique site-specific properties such as enhanced osteogenic differentiation, stemness, and anti-senescence properties compared to BMSCs from appendicular bone [9-11]. Although the roles of BMSCs underlying the age-related bone changes have been well characterized in axial and appendicular bones, however, the age-related properties of BMSCs from craniofacial bone and the associated mechanisms are still undefined.

Special AT-rich binding protein 2 (SATB2) is a nuclear matrix protein that functions as a key regulator of gene expression and chromatin remolding. Defects or haploinsufficiency of SATB2 in humans is a clinically recognizable syndrome characterized by intellectual disability, craniofacial abnormalities, dysmorphic features, cleft palate, micrognathia, and osteoporosis [12, 13]. SATB2 knockout mice exhibit craniofacial abnormalities that resemble those observed in humans carrying SATB2 gene mutations. These data strongly suggest that SATB2 is a multifunctional determinant of craniofacial patterning and osteoblast differentiation [14]. Our previous studies have revealed that SATB2 is critically involved in site-specific characteristics, such as osteogenesis, stemness and senescence of BMSCs from rat jaws [15]. Importantly, the induced pluripotent stem cells with SATB2 overexpression significantly enhanced bone regeneration and repair both in vitro and in vivo, underscoring its therapeutic potential for craniofacial bone regeneration [16]. However, the detailed molecular mechanisms underlying SATB2-mediated craniofacial regeneration are still in infancy. Savarese F and his colleague reported that forced SATB2 expression in embryonic stem cells antagonized differentiation-associated silencing of Nanog and enhanced the induction of Nanog [17]. Interestingly, Nanog plays a key role not only in the maintaining of ES cell pluripotency, but also reversing the proliferation and differentiation potential of older BMSCs [15, 17, 18]. Collectively, these findings prompted us to further investigate age-related properties of BMSCs from craniofacial bone and the roles of SATB2 underlying these properties.

Here, our data showed SATB2 diminished during age-related changes with the osteogenic, adipogenic, osteoclasts-activating potential, stemness, and senescence of human alveolar bone-derived BMSCs (AB-BMSCs). We further unraveled the pivotal roles of SATB2 in maintaining aforementioned biological characteristics by promoting Nanog transcription. Our study suggests the SATB2-Nanog axis is critically involved in age-related properties of BMSCs from craniofacial bone.

## RESULTS

### SATB2 expression in AB-BMSCs declines with age

**Figure 1:**
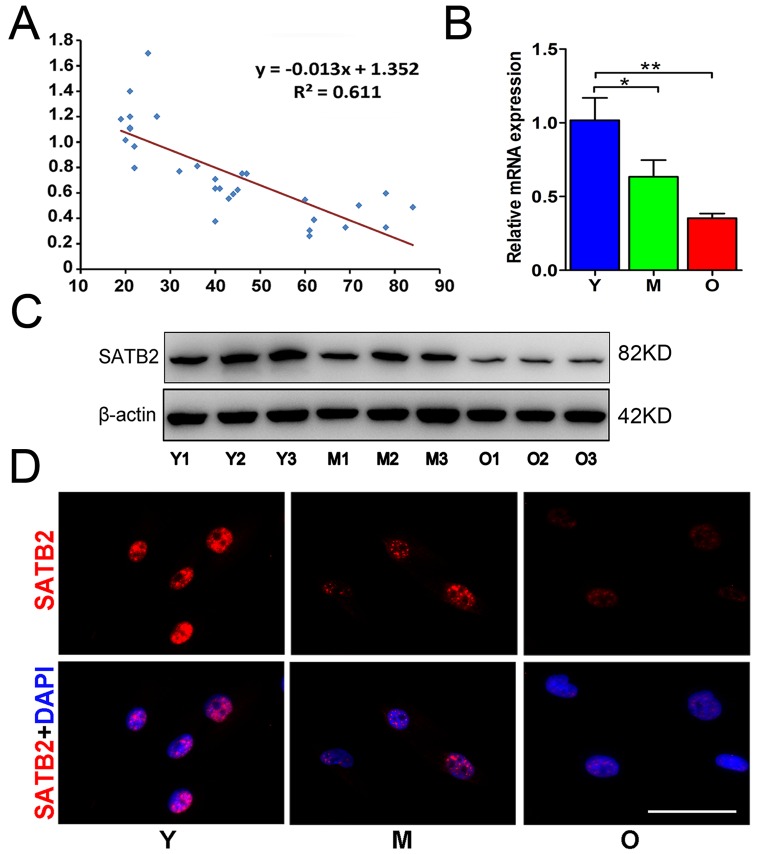
SATB2 expression in AB-BMSCs declines with age (**A**) Correlogram showed inverse correlation between SATB2 expression and age. SATB2 mRNA (**B**) and protein (**C**) levels decreased with age. (**D**) Immunofluorescence of AB-BMSCs in different ages demonstrated decreased SATB2 expression with aging. *p < 0.05, **p< 0.01. Scale bar=100μm.

To determine the change of SATB2 expression in AB-BMSCs with increased ages, AB-BMSCs from 30 healthy donors (19-84 years, mean age 43 ± 20 years) were utilized and subjected to real-time PCR assay. An inverse correlation between SATB2 abundance and age was found (R2 = 0.611; P = −0.013x + 1.352; Figure 1A). SATB2 mRNA levels in BMSC from older donors (O) were significantly lower as compared to middle (M) and young counterparts (Y) (Figure 1B). To further confirm this expression pattern of SATB2 with aging, three samples were randomly selected in Y, M and O groups and underwent western blot and immunofluorescence for SATB2 protein assessment. The results revealed a significant lower expression of SATB2 protein in M and O groups than Y group (Figure 1C and D). Taken together, these results indicate that SATB2 expression in AB-BMSCs is decreased with aging, thus suggesting potential roles of SATB2 underlying aging-related properties of alveolar bone and BMSCs.

### Age-related properties of AB-BMSCs

**Figure 2:**
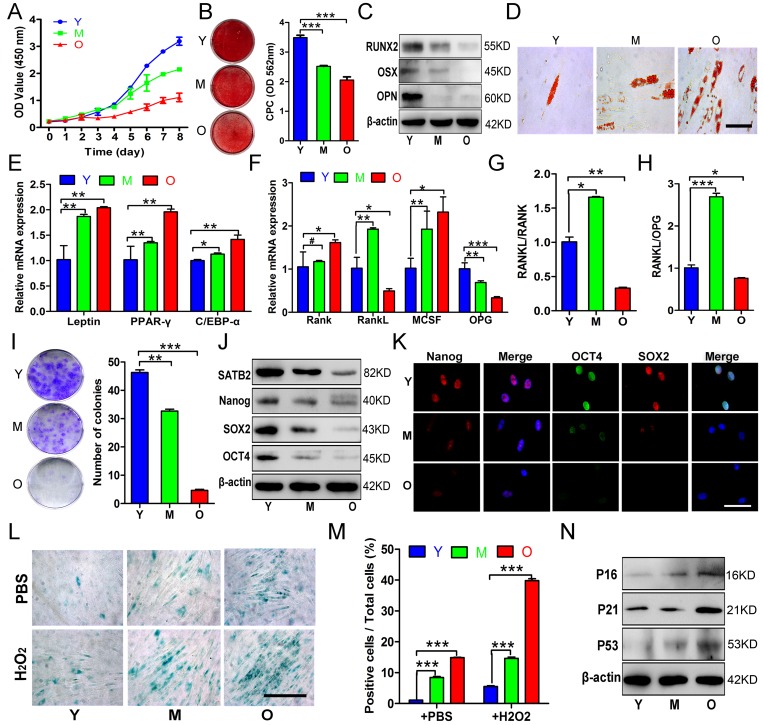
Age-related properties of AB-BMSCs (**A**) Decreased proliferation rates of BMSCs in group O as compared with Y, M groups was detected using CCK8 assay. (**B**, **C**) Decreased osteogenic differentiation of AB-BMSCs in M and O group in relative to Y group was observed by Alizarin red staining and osteogenic markers expression at day 14 after induction. (**D**, **E**) Enhanced adipogentic ability of AB-BMSCs associated with age as detected by oil red staining and higher mRNA levels of adipogenic markers at day 14 after induction. (**F**, **G**, **H**) The expression of RANK, RANKL, M-CSF and OPG, and the ratios of RANKL/RANK and RANKL/OPG were measured in different groups of AB-BMSCs as indicated. (**I**) More colony-forming units were observed in BMSCs from Y group than M and O group. (**J**, **K**) Western blot and immunofluorescence assays revealed decreased SATB2, Nanog, SOX2 and OCT4 protein expression associated with ages in different groups of AB-BMSCs as indicated. (**L**, **M**) SA-β-Gal staining revealed more senescent AB-BMSCs and the cells were easier to induce senescence under oxidative stress in M and O groups. (**N**) Western blot results showed higher P16, P21 and P53 expression in M and O groups compared to Y group. #p> 0.05, *p< 0.05, **p< 0.01, ***p< 0.001. Scale bar = 100 μm in K and Scale bar = 50 μm in D and L.

As anticipated, the proliferation rates, osteogenic potential of AB-BMSCs gradually decreased with aging (Figure 2A, B and C), while adipogenic potential enhanced with aging as indicated by increased lipid droplet formation and adipogenic markers expression (Figure 2D and E), generally in line with human femoral-derived BMSCs as previously reported [4]. Given that aging is not only associated with compromised osteogenesis, but also facilitates BMSCs-induced osteoclastogenesis through triggering expression of RANKL, macrophage-colony stimulation factor (M-CSF), and osteoprotegerin (OPG) [19]. Next, we examined the expression levels of these factors and found that both BMSCs from M and O groups exhibited higher M-CSF and lower OPG compared to Y group (Figure 2F). The RANKL expression and the ratios of RANKL/RANK, RANKL/OPG were increased in M group compared to Y group, but then decreased in O group (Figure 2F, G and H). These results implied that BMSCs from M group could be relatively easier to osteoclastogenesis induction, but this property tends to decrease with aging.

To understand these age-related properties in AB-BMSCs, we next compared the pluripotency and senescence properties of AB-BMSCs from young, middle, and older subjects. As shown in Fig. 2I, colony-forming units of AB-BMSCs significantly reduced with aging. In parallel with SATB2 decline, the expression of three master stemness factors Nanog, SOX2 and OCT4 was remarkably reduced with aging (Figure 2J and K). To explore the basal reactive oxygen species (ROS)-stressed senescence related to aging, we exposed AB-BMSCs to 60 μM hydrogen peroxide (H_2_O_2_) for 6 hours and then determine the cell senescence. As expected, more SA-β-Gal positive cells were observed in M and O groups than Y group (Figure 2L and M). Senescence-related factors P16, P21 and P53 showed markedly higher expression in M and O groups compared to Y group (Figure 2N). Together, these data indicate that declined pluripotency and increased senescence occurred in AB-BMSCs with aging.

### Gain and loss of function in SATB2 related to the properties of AB-BMSCs

**Figure 3:**
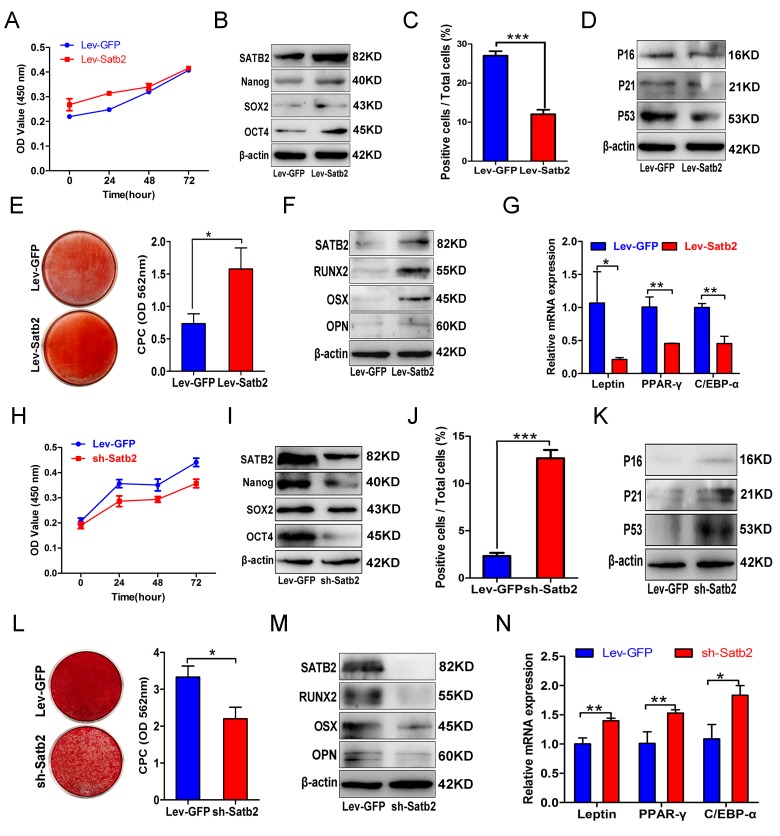
Gain and loss of function in SATB2 related to the properties of AB-BMSCs (**A**) Ectopic SATB2 overexpression in AB-BMSCs from O group had no significant influence on cell proliferation. (**B**) Upregulated SATB2, Nanog, SOX2 and OCT4 expression was observed in BMSCs transfected with SABT2 overexpression lentivirus. (**C**) SATB2 overexpression on AB-BMSCs from O group decreased the number of SA-β-Gal positive cells and downregulated P16, P21 and P53 expression as compared with cells infected with empty vector. (**D**, **E**, **F**) SATB2 overexpression on AB-BMSCs from O group enhanced osteogenic differentiation as demonstrated by stronger Alizarin red staining and higher SATB2, RUNX2, OSX and OPN expression as compared with control cells at day 14 after osteogenic induction. (**G**) The mRNA levels of adipogenic markers Leptin, PPAR-γ and C/EBP-α were decreased in SATB2 overexpression in AB-BMSCs from O group as compared with control cells at day 14 after adipogenic induction. (**H**) SATB2 knockdown in AB-BMSCs from Y group inhibited cell proliferation. (**I**) Downregulated SATB2, Nanog, SOX2 and OCT4 expression was detected upon endogenous SATB2 silencing. (**J**, **K**) Increased SA-β-Gal positive cell and upregulated P16, P21 and P53 expression were observed in SATB2 knockdown cells. (**L**, **M**) SATB2 knockdown in AB-BMSCs from Y group exhibited lower osteogenic differentiation as shown by weaker Alizarin red staining and lower SATB2, RUNX2, OSX and OPN expression as compared with control cells at day 14 after osteogenic induction. (**N**) The mRNA levels of adipogenic markers Leptin, PPAR-γ and C/EBP-α were increased in SATB2 knockdown in AB-BMSCs from Y group as compared with control cells at day 14 after adipogenic induction. *p< 0.05, **p < 0.01, ***p < 0.001. Scale bar = 100 μm.

To investigate whether SATB2 was involved in the age-related properties of AB-BMSCs, we employed the gain-of-function approach by introducing exogenous SATB2 into cells. AB-BMSCs from older subjects were transfected by exogenous SATB2 by lentiviral vector and followed by phenotypical change evaluation. Although cellular proliferation remained largely unaltered (Figure 3A), enforced SATB2 overexpression significantly enhanced pluripotency markers expression (Figure 3B), whereas reduced cellular senescence as reflected by reduced SA-β-Gal-positive cells and senescence marker expression (Figure 3C and D). Notably, ectopic SATB2 overexpression significantly promoted osteogenic differentiation of older AB-BMSCs and inhibited their adipogenic differentiation (Figure 3E, F and G). Taken together, these results suggest that SATB2 has the capability to reverse, at least partly, some aging-related phenotypes of older AB-BMSCs.

To verify the roles of SATB2 in governing the age-related properties of AB-BMSCs, endogenous SATB2 in AB-BMSCs from young donors was silenced by shRNA-mediated gene knockdown. The cellular proliferation rate significantly decreased after SATB2 knockdown (Figure 3H). In addition, SATB2 knock-down reduced the expression abundance of Nanog, SOX2 and OCT4 (Figure 3I), and increased cell senescence as shown by induced SA-β-Gal-positive cells and enhanced senescence markers expression (Figure 3J and K). Furthermore, SATB2 silencing remarkably inhibited osteogenic differentiation (Figure 3L and M) and promoted adipogenic differentiation of BMSCs (Figure 3N). Collectively, our results from both gain and loss of function experiments strongly support the idea that SATB2 is a critical regulator responsible for age-associated properties of AB-BMSCs.

### SATB2-modified BMSCs prevent alveolar bone loss during the aging of rats

**Figure 4:**
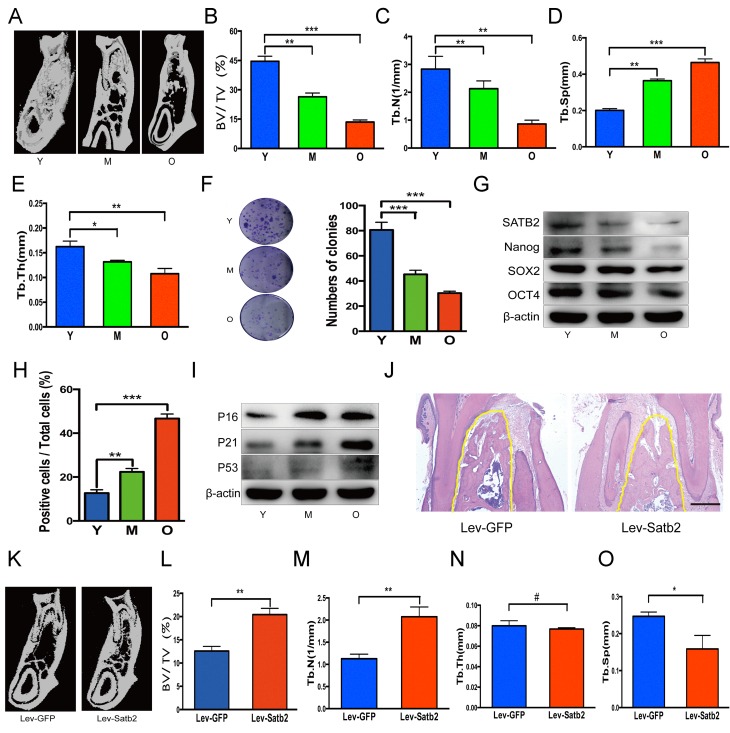
SATB2-modified BMSCs prevent alveolar bone loss of aging rats (**A**) The region of molar furcation in rats mandible demonstrated a significantly reduced bone mass with aging. (**B**) Bone histomorphometric parameters revealed a decrease in BV/TV, (**C**) Tb.N., (**D**) Tb.Th. and (**E**) an increase in Tb.Sp. (**F**) BMSCs from rats mandible showed a declined colony-forming units with aging. (**G**) Western blots showed the decrease of SATB2, Nanog, SOX2 and OCT4 expression with aging. (**H**) SA-β-Gal positive cells and senescence-related factors P16, P21 and P53 (I) increased with aging. (**J**) HE staining demonstrated abundant trabecular bone in Lev-satb2 group compared with Lev-GFP group (alveolar bone showed in yellow line). (**K**-**O**) Histomorphometric analysis of alveolar trabecular bone after treatment with BMSCs, representative micro-CT images (**K**), BV/TV (**L**), Tb.N. (**M**), Tb.Sp (**O**), and Tb.Th(N) between Lev-GFP group and Lev-satb2 group. #p> 0.05, *p< 0.05, **p< 0.01, ***p< 0.001. Scale bar = 100 μm.

To further understand the age-related properties of AB-BMSCs, we assessed the stemness and senescence of BMSCs and its potential correlation with the alveolar bone mass of rats at ages of 3, 12, and 18 months. We found that the alveolar bone mass in these rats decreased with aging (Figure 4A). Bone histomorpho-metric parameters revealed a decrease in BV/TV, Tb.N., Tb.Th. and an increase in Tb.Sp (Figure 4B, C, D and E). BMSCs from rats mandibular not only showed a declined colony-forming units with aging (Figure 4F), but also a reduced expression of stemness factors SATB2, Nanog, SOX2 and OCT4 (Figure 4G). More SA-β-Gal positive cells were observed with aging (Figure 4H), and senescence-related factors P16, P21 and P53 increased steadily in the aging of rats (Figure 4I). Of note, in rats, SATB2 expression in BMSCs also decreased with aging (Figure 4G).

To explore whether BMSCs rejuvenated by SATB2 could prevent alveolar bone loss during the aging, satb2-modified BMSCs therapy was implemented in rats at ages of 15 months. 3 months later, more abundant trabecular bone in Lev-satb2 group was demonstrated than in Lev-GFP group (Figure 4J). The results of three-dimensional micro-CT were consistent with histological staining, showing an increased bone mass (Figure 4K). Bone histomorphometric parameters demonstrated an increase in BV/TV, Tb.N., while a decrease in Tb.Sp (Figure 4L, M and O). There is no significant difference in Tb.Th between Lev-GFP group and Lev-satb2 group (Figure 4N). Together, these data suggest that AB-BMSCs derived from rats also demonstrate a declined pluripotency and increased senescence with aging, and transplantation of satb2- modified BMSCs could rejuvenate the alveolar bone during the aging of rats.

### SATB2 regulates age-related properties of AB-BMSCs by Nanog pathway

**Figure 5:**
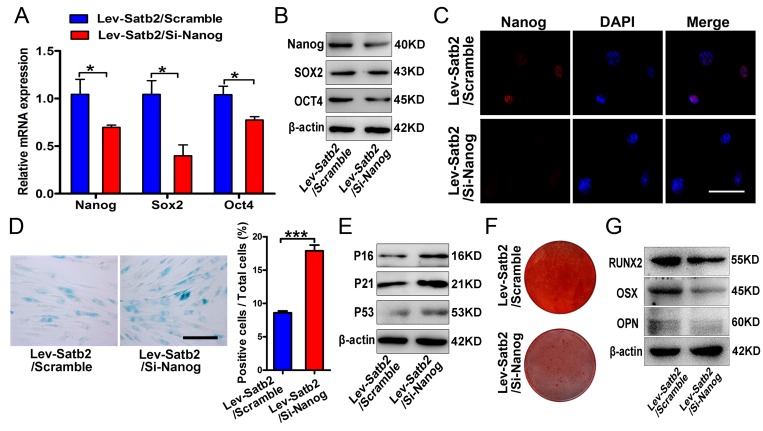
SATB2 regulated age-related properties of AB-BMSCs by Nanog pathway (**A**, **B**) Real-time PCR and western blot results showed the decrease of Nanog, SOX2 and OCT4 expressions after Nanog knockdown in AB-BMSCs with SATB2 overexpression (Lev-Satb2/Si-Nanog) in relative to control (Lev-Satb2/Scramble). (**C**) Immunofluorescence staining verified Nanog reduction upon Nanog knockdown mediated by siRNA. (**D**, **E**) Nanog knockdown increased the number of SA-β-Gal positive cells and P16, P21 and P53 abundance in AB-BMSCs with SATB2 overexpression. (**F**, **G**) Nanog knockdown largely abrogated the effects of SATB2 overexpression in AB-BMSCs from older donors as demonstrated by weaker Alizarin red staining and reduced RUNX2, OSX and OPN expression. *p< 0.05, ***p< 0.001. Scale bar = 100 μm.

Considering that SATB2 was intricately linked to senescence and pluripotency of AB-BMSCs, and the well-established regulatory role of Nanog in BMSCs stemness and senescence [18], so we next asked whether such effects of SATB2 on age-related properties of AB-BMSCs were mediated by Nanog and its associated downstream pathway. To address this, we knocked down Nanog in SATB2 stable overexpressing older AB-BMSCs and assessed the following phenotypic changes. Both mRNA and protein levels of Nanog, SOX2 and OCT4 were markedly decreased following Nanog knockdown in SATB2 overexpressing cells (Figure 5A, B and C). The number of SA-β-Gal positive cells significantly increased in parallel with P16, P21 and P53 upregulation (Figure 5D and E).

Importantly, Nanog knockdown inhibited osteogenic differentiation in AB-BMSCs with SATB2 over-expression (Figure 5F and G). Therefore, our results suggest that these age-related properties of AB-BMSCs might be associated with reduced expression of core pluripotency factors mediated by SATB2.

### SATB2 binds with nanog promoter and promotes its transcription in BMSCs

**Figure 6:**
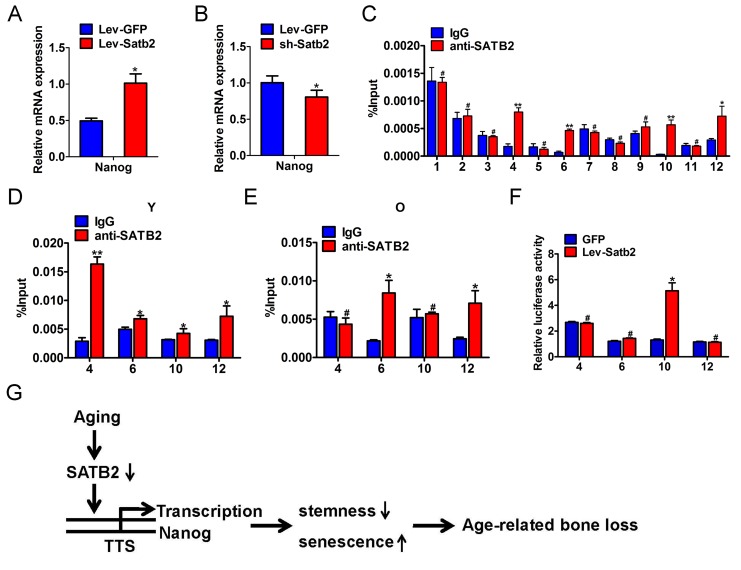
SATB2 binds to human Nanog promoter and promotes its transcription in AB-BMSCs The mRNA levels of Nanog were determined by real-time PCR upon SATB2 overexpression (**A**) and knockdown (**B**) in AB-BMSCS. (**C**) Twelve primers were designed to cover the human Nanog promoter region and used to identify binding sites of SATB2 by ChIP assay. Four putative SATB2-binding sites in Nanog promoter region were identified. The relative enrichment of these four putative binding sites of SATB2 in AB-BMSCs from young (**D**) and older donors (**E**). (**F**) Luciferase reporter assays indicated that the luciferase activity was significantly increased in reporter containing the binding site 10. (**G**) Schematic diagram illustrating the roles of SATB2-Nanog axis in aging-related properties of AB-BMSCs. #p> 0.05, *p< 0.05, **p< 0.01.

To investigate whether these core pluripotency factors were under direct or indirect regulation of SATB2 in AB-BMSCs, we performed the literature review and found that SATB2 binds the Nanog locus in vivo and contributes to the plasticity of Nanog expression and embryonic stem cell pluripotency [17]. No evidence has been found in direct binding between SATB2 and SOX2 or OCT4. However, the detailed molecular mechanisms underlying SATB2 regulatory roles on Nanog remain largely unexplored yet. Therefore, our following studies focused on SATB2 on Nanog transcription in BMSCs. Our data reveal that the level of Nanog transcript was induced upon SATB2 overexpression, and reduced following SATB2 knockdown (Figure 6A and B). These data suggest that Nanog expression might be regulated by SATB2 in a transcriptional manner. We reasoned that SATB2 could physically bind with the promoter of Nanog and promote its transcription in AB-BMSC. To address this, we performed the chromatin immuno-precipitation (ChIP) assays with specific antibody against human SATB2 and 12 primers covering the upstream −1850∼+350 of SATB2 (detailed sequences in Supplementary Table S3). Significant enrichment of SATB2 was identified in four putative binding sites in Nanog promoter region (Figure 6C, Supplementary Figure S2A and B). We next wonder whether such enrichments of SATB2 binding exist in AB-BMSCs from young and old subjects. Indeed, significant enrichments were found in all four binding sites in young AB-BMSCs and 2 sites in old AB-BMSCs, respectively (Figure 6D and E). The binding enrichment in site 4 and site 10 was significantly lower in old AB-BMSCs as compared to young counterparts. To further verify the regulatory function of SATB2 binding in Nanog transcription, we constructed the Nanog luciferase reporters which contained the four binding sites (each vector with one site) respectively. These reporters were co-transfected with human Satb2 plasmid into 293T cells. Results from luciferase reporter assays indicated that the luciferase activity was significantly increased in reporter containing the binding site 10, although the luciferase activates in the others were comparable with control (Figure 6F). Collectively, these findings support that SATB2 promotes Nanog transcription by directly binding to its promoter region in AB-BMSCs. Reduced SATB2 and its downstream mediator Nanog might be responsible for the aging-related properties in older AB-BMSC.

Taken together, based on our abovementioned data and previous literature, we propose that reduced SATB2 in aged AB-BMSCs was partially responsible for these age-related properties of AB-BMSCs. Nanog might be one of downstream effectors of SATB2 via direct transcriptional region underlying this biological process (Figure 6G).

## DISCUSSION

In this report, we firstly characterized age-related properties of BMSCs from human jaws, and found diminished SATB2 in AB-BMSCs with aging. Reduced SATB2 was critically involved in the age-related properties of BMSCs and Nanog was identified as a downstream effector of SATB2. Furthermore, our data revealed that SATB2 directly binding to Nanog promoter and in turn promoted its transcription in AB-BMSCs. Our findings extend the current understanding of the molecular mechanisms underlying the age-related properties of human craniofacial BMSCs.

Tissue or organ aging is a complex process characterized by the functional impairment of tissue maintenance, repair and regeneration [20]. This process might be caused by the age-associated decline in adult stem cell numbers and functions [21, 22], which leading to age-related bone loss throughout the body [3, 23, 24]. However, the intrinsic age-related changes of BMSCs from human craniofacial bone remains incompletely characterized. In this study, we characterized the alveolar-derived BMSCs from healthy subjects with different ages to reveal the phenotypic changes with aging. Consistent with previous reports about human femoral-derived BMSCs [4, 23], the proliferation and osteogenic differentiation potential of AB-BMSCs decreased with aging, whereas the senescence and adipogenic differentiation of AB-BMSCs increased. Interestingly, we also found that the osteoclast-inductive capacities of AB-BMSCs peaked in AB-BMSCs from middle aged donors but ultimately decreased with aging. This implied that AB-BMSCs-mediated bone remodeling may be the most active in early of elderly.

The core pluripotency transcription factors such as OCT4, SOX2 and Nanog play a pivotal role in self-renewal and properties maintenance of embryonic/adult stem cells [25, 26]. A line of evidence has indicated that these factors can reverse the age-related senescence of BMSCs and promote their differentiation potentials [18, 25, 27, 28]. Pluripotency decline may be the main intrinsic cause of stem cell aging and associated phenotypes such as reduced self-renewal and impaired differentiation potentials. Our findings showed protein levels of Nanog, OCT4, and SOX2 in AB-BMSCs decreased with aging, although it has been reported that Nanog, OCT4 mRNA expression in femoral-derived BMSCs is not affected by aging [23]. This discrepancy may be explained by phenotypical differences in site-specific BMSCs. Our studies and others have revealed that craniofacial BMSCs possess stronger stemness and anti-senescence properties compared to BMSCs from appendicular bone [9-11]. So, we reason that these core pluripotency transcription factors are critically involved in senescence-associated intrinsic mechanisms of craniofacial BMSCs. It may be a promising strategy by utilizing these pluripotency factors to reverse the aging, maintain stemness as well as block pre-mature senescence of BMSCs [15, 18, 29].

It is well established that SATB2 is a fundamental regulator underlying osteoblastogenesis and craniofacial bone formation [14, 30-32]. Our previous work showed that SATB2 could improve the stemness properties and anti-senescence capacity of BMSCs [15]. Moreover, induced pluripotent stem cells with SATB2 over-expression displayed enhanced osteogenic differen-tiation and bone formation both in vitro and in vivo [16]. As a transcription factor, SATB2 binds to specific DNA sequence, contributing to chromatin-remodeling, subnuclear DNA localization, and gene transcription [33-35]. In this study, by gain- and loss-of function experiments, we verified that SATB2 levels are closely associated with the age-related properties of AB-BMSCs, including declined stemness and osteogenic potential, and enhanced senescence and adipogenic differentiation potential. Enforced SATB2 can rejuvenate the older AB-BMSCs, while SATB2 knockdown promote senescence phenotype of young AB-BMSCs. These results highlight that SATB2 is critically involved in age-related property changes of AB-BMSCs. More importantly, we further identified Nanog as the downstream effector of SATB2 in AB-BMSCs based on the following findings. First, we found that Nanog expression was induced following SATB2 overexpression, while reduced upon SATB2 silencing in vitro. Then, endogenous Nanog knockdown largely abrogated the effects induced by SATB2 transduction in AB-BMSCs. These are also supported by the previous report in which they found that SATB2 binds the Nanog locus in vivo, and contribute to the plasticity of Nanog expression and mouse ES cell pluripotency. However, the detailed binding between SATB2 and Nanog locus has not been identified and functionally characterized thus far [17]. To resolve this, we analyzed the promoter region of human Nanog and identified four putative bind sites of SATB2. Moreover, different enrichment of SATB2 in Nanog promoter region in young and old AB-BMSCs might be responsible for reduced Nanog expression and age-related properties in old AB-BMSCs. Given the complex roles of SATB2 in diverse settings [14, 35, 36], here we can't rule out the possibility that other downstream mediators of SATB2 besides Nanog are involved in these age-related properties of AB-BMSC. It remains an open and interesting question to further explore the detailed mechanisms of SATB2 in mediating BMSCs aging.

How to efficiently reverse the aging process remain an unrealized challenge and a hot subject of intensive investigation [37]. Cell reprogramming by specific transcriptional factors is a novel therapeutic strategy with promising translational values as evidenced that iPS cells or stem/progenitor cells are successfully generated from differentiated cells by defined factors both in vitro and in vivo [38-40]. Recent evidence indicates that very small embryonic-like stem cells (VSELs) residing in adult tissues, which are a population of developmentally early stem cells, play a potential role in aging and organ rejuvenation [41]. Our findings that older BMSCs modified by exogenous SATB2 overexpression efficiently reverse the age-related properties suggest that therapeutic modification of SATB2 might be an alternative anti-aging approach to rejuvenate the aged BMSCs. However, more studies are needed to verify the translational values of SATB2 as a novel anti-aging target.

In conclusion, our data characterized the age-related properties of craniofacial BMSCs and revealed that decreased SATB2 expression of AB-BMSCs may be responsible for the age-related changes. Enforced SATB2 was able to rejuvenate the age-related properties of aged BMSCs. Mechanistically, SATB2 directly bind and transcriptionally activate Nanog gene expression in BMSCs. Further investigations into the mechanisms of SATB2 loss with aging in bone are warranted to expand our knowledge on BMSCs senescence as well as osteoporosis.

## MATERIALS AND METHODS

### Ethics statement

Investigation has been conducted in accordance with the ethical standards and according to the Declaration of Helsinki and according to national and international guidelines and has been approved by the Ethics and Research Committee of Nanjing Medical University.

### Cell culture

Trabecular bone of mandible was obtained from volunteer donors when they received impacted tooth extraction or dental implantation at our hospital. All volunteers were categorized into young group (Y, 19-27 years old), middle-aged group (M, 32-47 years old) and old group (O, 60-84 years old) according to age. Informed consent was obtained before volunteers enrolled in this study. Human BMSCs from mandibular bones were obtained according to previous methods [10, 42]. BMSCs were cultured in 25cm2 cell culture flasks with medium consisting of Dulbecco's modified Eagle's medium (DMEM) (Gibco Life Technologies, NY, USA), 100 U/ml penicillin and 100 μg/ml streptomycin, 10% fetal bovine serum (FBS) (Hyclone, Logan, UT, USA) at 37°C maintained in 5% CO2. BMSCs were confirmed by positive expression of MSC markers CD29, CD 90, CD105 and negative expression of hematopoietic markers CD34, CD45 (Supplementary Figure S1).

### Western blots and real-time PCR

Western blots and real-time PCR analysis were performed as our previous reports [43]. The primary antibodies used for western blots are listed in Supplementary Table S2. Western blots results were quantified with Quantity One software (Bio-Rad Laboratories, Calif., USA). The primer sequences used for Real-time PCR are listed in Supplementary Table S2. The expression levels of each mRNA were normalized to GAPDH.

### Immunofluorescence

Cells were firstly permeabilized with 0.2% Triton X-100 (Sigma, St. Louis, Mo., USA) for 10 min and followed by incubation with primary antibodies at 4°C overnight. Cells and tissues were then incubated with FITC-tagged or TRITC-tagged secondary antibodies for 60 min. The nuclei were stained with DAPI (Beyotime Institute of Biotechnology, Haimen, China) and immunofluorescent images were captured with Leica DM4000 (Leica Microsystems, Mannheim, Germany). Cell Proliferation and Colony-forming Assays. Cell proliferation ability was measured with CCK8 kit (Dojindo, Kamimashiki-gun, Kumamoto, Japan) assay. Briefly, cells were prepared into 96-well plates at a cellular density of 1 × 103 cells/well. A 1:10 diluted CCK8 solution in DMEM was added to the cells. After 2 hours incubation at 37°C, the results were measured by automatic enzyme-linked immunosorbent assay reader (BioTek Instruments Ins., USA) at 450 nm. Cell colony-forming assay were performed as we described previously [44].

### Osteogenic induction and Alizarin Red staining

Osteogenic differentiation of BMSCs was induced using osteogenic media containing supplements of 10-7 M dexamethasone (Sigma), 10 mM β-glycerophosphate (Sigma) and 50 μg/ml ascorbic acid (Sigma). Following 14 days of osteogenic induction, cells were fixed in anhydrous alcohol for 30 min followed by staining with 2% Alizarin Red S (PH 4.2) (Sigma). The calcium accumulations were quantified with cetylpyridinium chloride (CPC) and measured by automatic enzyme-linked immunosorbent assay reader (BioTek instruments Tnc., USA) at 510 nm.

### Adipogenic induction and Oil Red staining

Adipogenic differentiation of BMSCs was induced adipogenic media containing supplements of 10-6 M dexamethasone (Sigma), 10 μg/ml insulins (Sigma), 0.5 mM 3-isobutyl-1-methylxanthine (Sigma) and 0.2 mM indomethacin (Sigma). Following adipogenic induction for 14 days, the cells were fixed with 4% paraformal-dehyde for 10 min and stained with Oil Red O. The images were captured by Leica DMI3000B (Leica). Lipid droplet areas were analyzed by Image J software (National Institutes of Health, USA).

### Cell senescence-associated β-galactosidase staining

Senescent cells were determined by senescence-associated-β-galactosidase (SA-β-Gal) activity, which was identified by β-Gal Staining Kit (GenMed, USA) according to the manufacturer's instructions. The images were captured by Leica DMI3000B (Leica) and senescent cells were also quantified by Image J software.

### Animal experiments

All procedures were carried out according to the guidelines of the Animal Care Committee of Nanjing Medical University. As we described previously [43], BMSCs derived from mandibular were isolated from young group (3 months old), middle-aged group (12 months old) and old group (18 months old) male Sprague-Dawley (SD) rats. 15 months old SD rats were transplanted with green fluorescent protein (GFP) modified or SATB2 enforced BMSCs (1 × 107) by tail vein at week 2, 6, and 12 after micro-CT scan. Rats were killed at week 14 (n=5 per group).

### Micro-CT measurement

The micro-structure of the first molar furcation in mandible was evaluated using a micro-CT system (Skyscan 1176, Kontich, Belgium). The trabecular bone was scanned at high resolution (18 μm) and energy of 50 kV and 456 μA. NRecon v1.6 and CTAn v1.13.8.1 software were used to reconstruct and analyze the 3D images. To analyze the bone microarchitecture, 4 parameters were calculated: bone volume ratio (BV/TV, %), trabecular thickness (Tb.Th.), trabecular number (Tb.N.), and trabecular separation (Tb.Sp.)

### Histological observation

Mandibular bone specimens were fixed with 4% paraformaldehyde for 24 hours and decalcified in a solution of 10% EDTA (pH7.4). Then, bone specimens embedded in paraffin wax, sectioned into 4 μm-thick slices, and placed on adhesion microscope slides. The trabecular bone of the furcation area was observed by hematoxylin and eosin (HE) staining.

### Chromatin immunoprecipitation (ChIP)

For ChIP assays, different primers were synthetized and covered −1850 to +350 of Nanog promoter. BMSCs were cross-linked with 1% formaldehyde at 37°C for 20 min. The cells were then lysed in SDS buffer and sonicated to shear DNA. ChIP analysis was carried out using EZ-ChIP (Millipore Corporation, Billerica, Mass., USA) according to the manufacturer's protocol. Lysates diluted with ChIP dilution buffer were immuno-precipitated with anti-SATB2 (Proteintech, Chicago, USA) or rabbit IgG as an internal control. The precipitated DNA was analyzed by real-time PCR and semi-quantitative PCR.

### Dual luciferase reporter assay

The putative binding region of SATB2 in human Nanog promoter were cloned downstream of the firefly luciferase gene (FL) in pGL3-basic luciferase reporter vector (Promega, USA). For luciferase reporter assays, 293T cells were co-transfected with individual pGL3-Nanog reporter and Lev-Satb2 plasmids. After 48 hours transfection, cells lysates were collected and assayed with the Dual-Luciferase Assay (Promega) following the manufacturer's instructions. The pRL Renilla luciferase (RL) reporter was used for data normalization. The results were displayed as the ratio of FL/RL activity.

### Statistical analysis

All in vitro experiments were repeated independently at least three times. Results were presented as mean ± standard deviation (SD). The statistical significance between groups was calculated using Student's t-test or ANOVA analysis as indicated. P-value < 0.05 (two sides) was considered significant.

### SUPPLEMENTARY MATERIAL FIGURES AND TABLES



## References

[1] Teitelbaum SL (2010). Stem cells and osteoporosis therapy. Cell Stem Cell.

[2] Bidwell JP, Alvarez MB, Hood M, Childress P (2013). Functional impairment of bone formation in the pathogenesis of osteoporosis: the bone marrow regenerative competence. Curr Osteoporos Rep.

[3] Yao W, Guan M, Jia J, Dai W, Lay YA, Amugongo S, Liu R, Olivos D, Saunders M, Lam KS, Nolta J, Olvera D, Ritchie RO, Lane NE (2013). Reversing bone loss by directing mesenchymal stem cells to bone. Stem Cells.

[4] Zhou S, Greenberger JS, Epperly MW, Goff JP, Adler C, Leboff MS, Glowacki J (2008). Age-related intrinsic changes in human bone-marrow-derived mesenchymal stem cells and their differentiation to osteoblasts. Aging Cell.

[5] Jiang SS, Chen CH, Tseng KY, Tsai FY, Wang MJ, Chang IS, Lin JL, Lin S (2011). Gene expression profiling suggests a pathological role of human bone marrow-derived mesenchymal stem cells in aging-related skeletal diseases. Aging (Albany NY).

[6] Drozdzowska B, Pluskiewicz W, Michno M (2006). Tooth count in elderly women in relation to their skeletal status. Maturitas.

[7] Erdogan O, Incki KK, Benlidayi ME, Seydaoglu G, Kelekci S (2009). Dental and radiographic findings as predictors of osteoporosis in postmenopausal women. Geriatr Gerontol Int.

[8] Chai Y, Maxson RE (2006). Recent advances in craniofacial morphogenesis. Dev Dyn.

[9] Aghaloo TL, Chaichanasakul T, Bezouglaia O, Kang B, Franco R, Dry SM, Atti E, Tetradis S (2010). Osteogenic potential of mandibular vs. long-bone marrow stromal cells. J Dent Res.

[10] Akintoye SO, Lam T, Shi S, Brahim J, Collins MT, Robey PG (2006). Skeletal site-specific characterization of orofacial and iliac crest human bone marrow stromal cells in same individuals. Bone.

[11] Dong W, Ge J, Zhang P, Fu Y, Zhang Z, Cheng J, Jiang H (2014). Phenotypic characterization of craniofacial bone marrow stromal cells: unique properties of enhanced osteogenesis, cell recruitment, autophagy, and apoptosis resistance. Cell Tissue Res.

[12] Zarate YA, Perry H, Ben-Omran T, Sellars EA, Stein Q, Almureikhi M, Simmons K, Klein O, Fish J, Feingold M, Douglas J, Kruer MC, Si Y (2015). Further supporting evidence for the SATB2-associated syndrome found through whole exome sequencing. Am J Med Genet A.

[13] Döcker D, Schubach M, Menzel M, Munz M, Spaich C, Biskup S, Bartholdi D (2014). Further delineation of the SATB2 phenotype. Eur J Hum Genet.

[14] Dobreva G, Chahrour M, Dautzenberg M, Chirivella L, Kanzler B, Fariñas I, Karsenty G, Grosschedl R (2006). SATB2 is a multifunctional determinant of craniofacial patterning and osteoblast differentiation. Cell.

[15] Dong W, Zhang P, Fu Y, Ge J, Cheng J, Yuan H, Jiang H (2015). Roles of SATB2 in site-specific stemness, autophagy and senescence of bone marrow mesenchymal stem cells. J Cell Physiol.

[16] Ye JH, Xu YJ, Gao J, Yan SG, Zhao J, Tu Q, Zhang J, Duan XJ, Sommer CA, Mostoslavsky G, Kaplan DL, Wu YN, Zhang CP (2011). Critical-size calvarial bone defects healing in a mouse model with silk scaffolds and SATB2-modified iPSCs. Biomaterials.

[17] Savarese F, Dávila A, Nechanitzky R, De La Rosa-Velazquez I, Pereira CF, Engelke R, Takahashi K, Jenuwein T, Kohwi-Shigematsu T, Fisher AG, Grosschedl R (2009). Satb1 and Satb2 regulate embryonic stem cell differentiation and Nanog expression. Genes Dev.

[18] Han J, Mistriotis P, Lei P, Wang D, Liu S, Andreadis ST (2012). Nanog reverses the effects of organismal aging on mesenchymal stem cell proliferation and myogenic differentiation potential. Stem Cells.

[19] Cao JJ, Wronski TJ, Iwaniec U, Phleger L, Kurimoto P, Boudignon B, Halloran BP (2005). Aging increases stromal/osteoblastic cell-induced osteoclastogenesis and alters the osteoclast precursor pool in the mouse. J Bone Miner Res.

[20] Zhao B, Benson EK, Qiao R, Wang X, Kim S, Manfredi JJ, Lee SW, Aaronson SA (2009). Cellular senescence and organismal ageing in the absence of p21(CIP1/WAF1) in ku80(−/−) mice. EMBO Rep.

[21] Janzen V, Forkert R, Fleming HE, Saito Y, Waring MT, Dombkowski DM, Cheng T, DePinho RA, Sharpless NE, Scadden DT (2006). Stem-cell ageing modified by the cyclin-dependent kinase inhibitor p16INK4a. Nature.

[22] Noda S, Horiguchi K, Ichikawa H, Miyoshi H (2008). Repopulating activity of ex vivo-expanded murine hematopoietic stem cells resides in the CD48-c-Kit+Sca-1+lineage marker- cell population. Stem Cells.

[23] Siegel G, Kluba T, Hermanutz-Klein U, Bieback K, Northoff H, Schäfer R (2013). Phenotype, donor age and gender affect function of human bone marrow-derived mesenchymal stromal cells. BMC Med.

[24] Kassem M, Marie PJ (2011). Senescence-associated intrinsic mechanisms of osteoblast dysfunctions. Aging Cell.

[25] Johansson H, Simonsson S (2010). Core transcription factors, Oct4, Sox2 and Nanog, individually form complexes with nucleophosmin (Npm1) to control embryonic stem (ES) cell fate determination. Aging (Albany NY).

[26] Kashyap V, Rezende NC, Scotland KB, Shaffer SM, Persson JL, Gudas LJ, Mongan NP (2009). Regulation of stem cell pluripotency and differentiation involves a mutual regulatory circuit of the NANOG, OCT4, and SOX2 pluripotency transcription factors with polycomb repressive complexes and stem cell microRNAs. Stem Cells Dev.

[27] Liu TM, Wu YN, Guo XM, Hui JH, Lee EH, Lim B (2009). Effects of ectopic Nanog and Oct4 overexpression on mesenchymal stem cells. Stem Cells Dev.

[28] Go MJ, Takenaka C, Ohgushi H (2008). Forced expression of Sox2 or Nanog in human bone marrow derived mesenchymal stem cells maintains their expansion and differentiation capabilities. Exp Cell Res.

[29] Squillaro T, Severino V, Alessio N, Farina A, Di Bernardo G, Cipollaro M, Peluso G, Chambery A, Galderisi U (2015). De-regulated expression of the BRG1 chromatin remodeling factor in bone marrow mesenchymal stromal cells induces senescence associated with the silencing of NANOG and changes in the levels of chromatin proteins. Cell Cycle.

[30] Toti P, Sbordone C, Martuscelli R, Califano L, Ramaglia L, Sbordone L (2013). Gene clustering analysis in human osteoporosis disease and modifications of the jawbone. Arch Oral Biol.

[31] Leoyklang P, Suphapeetiporn K, Siriwan P, Desudchit T, Chaowanapanja P, Gahl WA, Shotelersuk V (2007). Heterozygous nonsense mutation SATB2 associated with cleft palate, osteoporosis, and cognitive defects. Hum Mutat.

[32] Zhang J, Tu Q, Grosschedl R, Kim MS, Griffin T, Drissi H, Yang P, Chen J (2011). Roles of SATB2 in osteogenic differentiation and bone regeneration. Tissue Eng Part A.

[33] Zhou LQ, Wu J, Wang WT, Yu W, Zhao GN, Zhang P, Xiong J, Li M, Xue Z, Wang X, Xie XM, Guo ZC, Lv X, Liu DP (2012). The AT-rich DNA-binding protein SATB2 promotes expression and physical association of human (G)γ- and (A)γ-globin genes. J Biol Chem.

[34] Dobreva G, Dambacher J, Grosschedl R (2003). SUMO modification of a novel MAR-binding protein, SATB2, modulates immunoglobulin mu gene expression. Genes Dev.

[35] Gyorgy AB, Szemes M, de Juan Romero C, Tarabykin V, Agoston DV (2008). SATB2 interacts with chromatin-remodeling molecules in differentiating cortical neurons. Eur J Neurosci.

[36] Shin EA, Sohn EJ, Won G, Yun S, Kim J, Kim SH (2016). SATB2 is localized to the centrosome and spindle maintenance and its knockdown leads to down-regulation of CDK2. In Vitro Cell Dev Biol Anim.

[37] Conboy IM, Conboy MJ, Rebo J (2015). Systemic Problems: A perspective on stem cell aging and rejuvenation. Aging (Albany NY).

[38] Takahashi K, Tanabe K, Ohnuki M, Narita M, Ichisaka T, Tomoda K, Yamanaka S (2007). Induction of pluripotent stem cells from adult human fibroblasts by defined factors. Cell.

[39] Huang P, Zhang L, Gao Y, He Z, Yao D, Wu Z, Cen J, Chen X, Liu C, Hu Y, Lai D, Hu Z, Chen L (2014). Direct reprogramming of human fibroblasts to functional and expandable hepatocytes. Cell Stem Cell.

[40] Ohmine S, Squillace KA, Hartjes KA, Deeds MC, Armstrong AS, Thatava T, Sakuma T, Terzic A, Kudva Y, Ikeda Y (2012). Reprogrammed keratinocytes from elderly type 2 diabetes patients suppress senescence genes to acquire induced pluripotency. Aging (Albany NY).

[41] Ratajczak MZ, Shin DM, Liu R, Mierzejewska K, Ratajczak J, Kucia M, Zuba-Surma EK (2012). Very small embryonic/epiblast-like stem cells (VSELs) and their potential role in aging and organ rejuvenation--an update and comparison to other primitive small stem cells isolated from adult tissues. Aging (Albany NY).

[42] Mason S, Tarle SA, Osibin W, Kinfu Y, Kaigler D (2014). Standardization and safety of alveolar bone-derived stem cell isolation. J Dent Res.

[43] Zhang P, Men J, Fu Y, Shan T, Ye J, Wu Y, Tao Z, Liu L, Jiang H (2012). Contribution of SATB2 to the stronger osteogenic potential of bone marrow stromal cells from craniofacial bones. Cell Tissue Res.

[44] Ge J, Guo S, Fu Y, Zhou P, Zhang P, Du Y, Li M, Cheng J, Jiang H (2015). Dental Follicle Cells Participate in Tooth Eruption via the RUNX2-MiR-31-SATB2 Loop. J Dent Res.

