# Supplementary material for: SATB2-Nanog axis links age-related intrinsic changes of mesenchymal stem cells from craniofacial bone
Source: Aging (Albany NY). 2016 Sep 14;8(9):2006–19. doi: 10.18632/aging.101041 (PMC5076449; doi:10.18632/aging.101041)
Supplement: Supplementary file 1 [file aging-08-2006-s001.pdf]

SUPPLEMENTARY MATERIAL

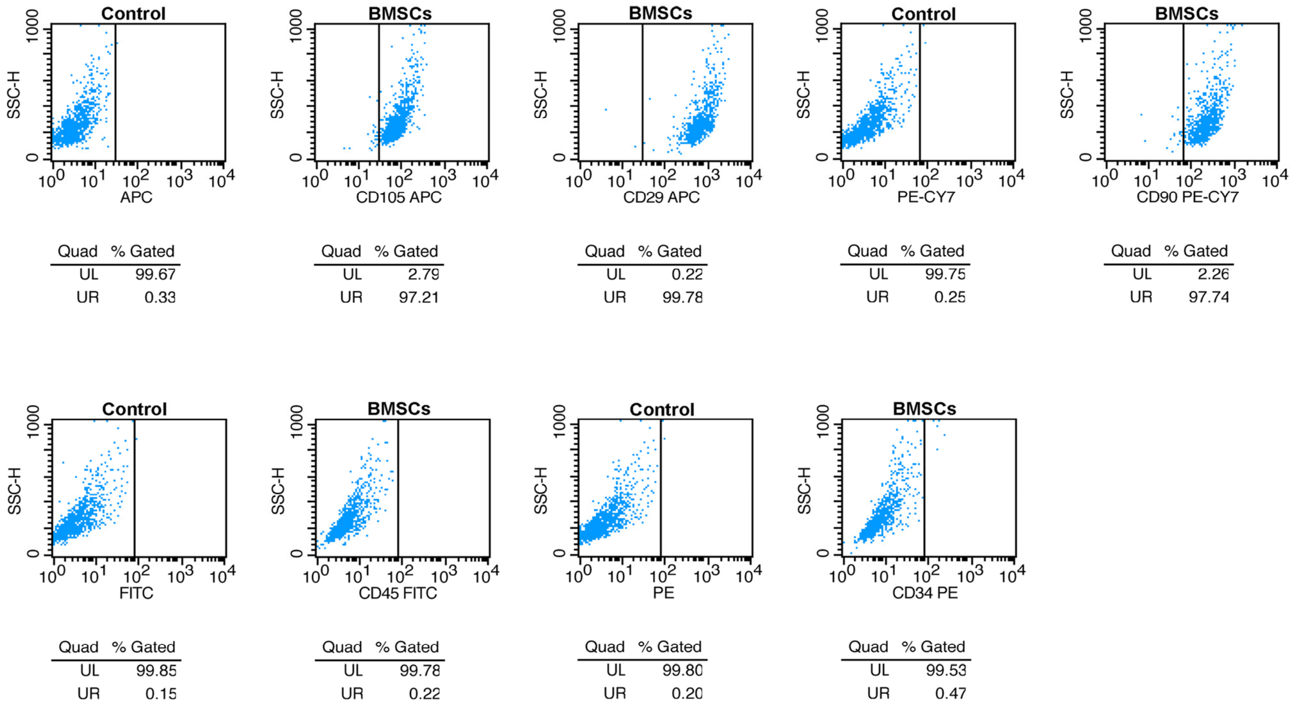

Supplementary Figure S1. BMSCs were confirmed by flow cytometry.

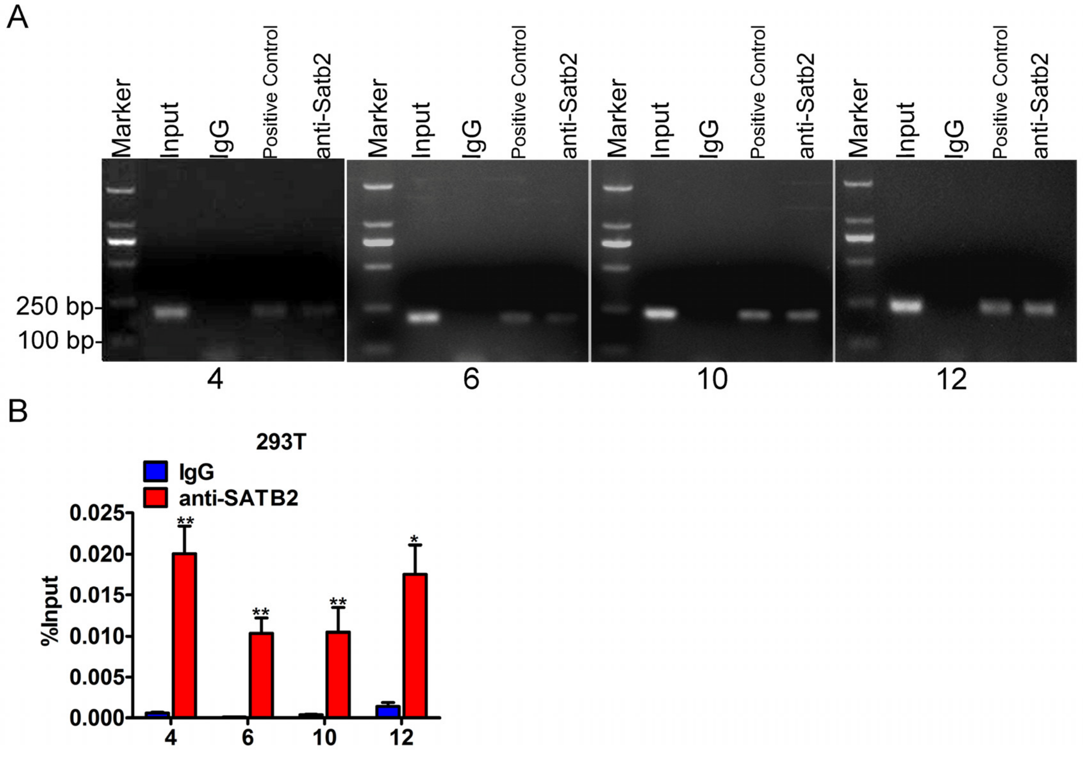

Supplementary Figure S2. (**A**) ChIP assays showed four putative binding sites among 12 primers covering the upstream -1850~+350 of SATB2 in human Nanog promoter region in BMSCs. (**B**) The four positive binding sites were further proved in 293T cells by real-time PCR. *p < 0.05, **p < 0.01.

Table S1. Antibodies used in Western blot

Antibody	Dilution	Source	Code number
SATB2	1:800	Abcam	Ab69995
NANOG	1:400	Santa Cruz	SC-33760
SOX2	1:400	Santa Cruz	SC-17320
OCT4	1:400	Santa Cruz	SC-5279
RUNX2	1:1000	Cell Signaling Technology	#12556
OSX	1:800	Abcam	Ab22552
OPN	1:800	Bioworld	BS1264
P16	1:800	Abcam	Ab51243
P21	1:500	Proteintech	10355-1-AP
P53	1:500	Proteintech	10442-1-AP
β-actin	1:800	Boisynthesis Biotechnology	Bs-oo61R

Table S2. Sequence information on specific primers used in this study

Gene	Primer Sequence (5' - 3')	Product size (bp)	Tm (˚C)
Satb2	F: 5'- CCAGGAGTTTGGGAGATGGTAT - 3'<br>R: 5'- GTGAGGAGACTGTTCGTTGGTT - 3'	101	60.0
Nanog	F: 5'- AAGGTCCCGGTCAAGAAACAG - 3'<br>R: 5'- CTTCTGCGTCACACCATTGC - 3'	126	59.7
Sox2	F: 5'- GGCAGAGAAGAGAGTGTTTGC - 3'<br>R: 5'- GCCGCCGATGATTGTTATT - 3'	120	59.8
Oct4	F: 5'- TGAGAGGCAACCTGGAGAAT - 3'<br>R: 5'- AACCACACTCGGACCACATC - 3'	115	60.0
OPG	F: 5'- GCGCTCGTGTTTCTGGACATC-3'<br>R: 5'- CCAGGAGGACATTTGTCACACAAC - 3'	149	61.9
RANK	F: 5'- GAGGCACCAGAGTTAGTCTGC - 3'<br>R: 5'- GGAGGTGTTCACGTTGAGAATC - 3'	101	61.9
RANKL	F: 5'- TGCCAGTGGGAGATGTTAGAC - 3'<br>R: 5'- CCTTCAATTGCGCTAGATGAC - 3'	119	60.0
M-CSF	F: 5'- TAGCCACATGATTGGGAGTGGA - 3'<br>R: 5'- CTCAAATGTAATTTGGCACGAGGTC - 3'	122	60.0
Leptin	F: 5'- AGTTGTACTTCCAGTGCGTCTC - 3'<br>R: 5'- TACTTTCACAGTCGGGTT - 3'	130	59.8

PPAR-γ	F: 5’- AGCTGAACCACCCTGAGTCC - 3’<br>R: 5’- TCATGTCTGTCTCCGTCTTCTTG - 3’	125	59.9
C/EBP-α	F: 5’- CACGAAGCACGATCAGTCCAT - 3’<br>R: 5’- CGCACATTCACATTGCACAAG - 3’	124	59.8
GAPDH	F: 5’- GGAGATTACTGCCCTGGCTCCTA - 3<br>R: 5’- GACTCATCGTACTCCTGCTTGCTG - 3’	178	60.0

Table S3. Sequence of the 12 ChIP primers. Red represents four positive binding sites

**Number**		**Primers**	**Sites**
Nanog1	F<br>R	CATTTTAACACATCCTTAGTCCAGC<br>GCTCCCTGTCCCATTGTGT	**`-1850～-1663**
Nanog2	F<br>R	AATGGGACAGGGAGCGG<br>GAAATAGGATGATTTCTTTAAAAGAAGG	**`-1677～-1453**
Nanog3	F<br>R	AAGAAATCATCCTATTTCCTACGAGA<br>TATTGCTTGATGTGAAACAAGGA	**`-1470～-1301**
**Nanog4**	**F**<br>**R**	**AGGAGCAGAGTGCAGAGGAG**<br>**CTTCCTATTCCCAAACCCAAC**	**`-1226～-1076**
Nanog5	F<br>R	GGGTTTGGGAATAGGAAGGA<br>GTGGGTGTGTGTGTTTCTGG	**`-1093～-965**
**Nanog6**	**F**<br>**R**	**CCATAAATGTTAGTGCTGGAACC**<br>**AGTTTGGTTTCTTGTCTATCCCTC**	**`-1050～-838**
Nanog7	F<br>R	AAAGGAAACTAAGGTAGGTGCTGA<br>CATGCACCTTAAATTCCTGAGG	**`-836～-601**
Nanog8	F<br>R	CAGGAATTTAAGGTGCATGC<br>GAGTTTGAAACCAGCCTGGC	**`-619～-366**
Nanog9	F<br>R	TGGCCAGGCTGGTTTC<br>CCAGCAACAAATACTTCTAGGTTC	**`-387～-186**
**Nanog10**	**F**<br>**R**	**GCTGGGTTTGTCTTCAGGTT**<br>**TCCCGTCTACCAGTCTCACC**	**`-190～-79**
Nanog11	F<br>R	AAATCTTTGTTAAATTTTTGGTTGG<br>AAGCTGGAGTGCAGTGGTGT	**`+1～+200**
**Nanog12**	**F**<br>**R**	**GGTAGGTGGAGGTTGTAGTGAG**<br>**TGCACATGTACAATGCACGT**	**`+151～+350**
